# Internet Cognitive Behavioral Therapy for Women With Postnatal Depression: A Randomized Controlled Trial of MumMoodBooster

**DOI:** 10.2196/jmir.4993

**Published:** 2016-03-07

**Authors:** Jeannette Milgrom, Brian G Danaher, Alan W Gemmill, Charlene Holt, Christopher J Holt, John R Seeley, Milagra S Tyler, Jessica Ross, Jennifer Ericksen

**Affiliations:** ^1^Parent-Infant Research InstituteMelbourneAustralia; ^2^University of MelbourneMelbourne School of Psychological SciencesMelbourneAustralia; ^3^Austin HealthDepartment of Clinical and Health PsychologyMelbourneAustralia; ^4^Oregon Research InstituteEugene, ORUnited States

**Keywords:** postnatal depression, postpartum depression, cognitive behavioral therapy, Internet-based intervention, randomized controlled trial

## Abstract

**Background:**

There are few published controlled trials examining the efficacy of Internet-based treatment for postnatal depression (PND) and none that assess diagnostic status (clinical remission) as the primary outcome. This is despite the need to improve treatment uptake and accessibility because fewer than 50% of postnatally depressed women seek help, even when identified as depressed.

**Objective:**

In a randomized controlled trial (RCT), we aimed to test the efficacy of a 6-session Internet intervention (the MumMoodBooster program, previously evaluated in a feasibility trial) in a sample of postnatal women with a clinical diagnosis of depression. The MumMoodBooster program is a cognitive behavioral therapy (CBT) intervention, is highly interactive, includes a partner website, and was supported by low-intensity telephone coaching.

**Methods:**

This was a parallel 2-group RCT (N=43) comparing the Internet CBT treatment (n=21) to treatment as usual (n=22). At baseline and at 12 weeks after enrollment, women’s diagnostic status was assessed by telephone with the Standardized Clinical Interview for DSM-IV (SCID-IV) and symptom severity with the Beck Depression Inventory (BDI-II). Depression symptoms were measured repeatedly throughout the study period with the Patient Health Questionnaire (PHQ-9).

**Results:**

At the end of the study, 79% (15/19) of women who received the Internet CBT treatment no longer met diagnostic criteria for depression on the SCID-IV (these outcome data were missing for 2 intervention participants). This contrasted with only 18% (4/22) remission in the treatment as usual condition. Depression scores on the BDI-II showed a large effect favoring the intervention group (*d*=.83, 95% CI 0.20-1.45). Small to medium effects were found on the PHQ-9 and on measures of anxiety and stress. Adherence to the program was very good with 86% (18/21) of users completing all sessions; satisfaction with the program was rated 3.1 out of 4 on average.

**Conclusions:**

Our results suggest that our Internet CBT program, MumMoodBooster, is an effective treatment option for women clinically diagnosed with PND. This is one of only two controlled evaluations of specialized online psychological treatment among women clinically diagnosed with PND. MumMoodBooster appears to be a feasible, effective treatment option, which is potentially accessible to large numbers of women in metropolitan, rural, and remote areas. Future work might be focused profitably on establishing comparability with face-to-face treatments and purely self-guided delivery. We have commenced a larger RCT comparing MumMoodBooster with face-to-face CBT.

**Trial Registration:**

Australian and New Zealand Clinical Trials Registry (ANZCTR): ACTRN12613000113752; https://www.anzctr.org.au/Trial/Registration/TrialReview.aspx?id=363561 (Archived by WebCite® at http://www.webcitation.org/6f64kuyLf).

## Introduction

Approximately 13% of women suffer postnatal depression (PND) by the time their baby is 3 months old [1]. PND, defined as an episode of major or minor depression beginning in the first year postbirth, lies between “baby blues” and postpartum psychosis in severity and prevalence. Left untreated, PND has significant deleterious effects on the mother (her mental health, her relationship with her infant, her potentially suicidal behavior), her family (interrupted employment, partner’s mental health, relationship problems), and her child’s development [2-4]. Yet despite the existence of effective treatments for depression, fewer than 50% of postnatal women seek or accept help, even when identified as depressed [5,6]. Barriers to help seeking and treatment uptake include perceived stigma, fear of being judged a “bad mother,” lowered motivation toward help seeking due to symptoms (eg, fatigue, feelings of hopelessness), concerns about medication while breastfeeding, unequal availability of clinic-based services in remote and rural areas, and logistical difficulties in attending face-to-face clinic counseling with a young infant [7-9]. Further, for most postnatal women, psychotherapy is preferred to pharmacotherapy in the treatment of mental health difficulties often due to concerns over breastfeeding [10]. As pointed out in previous work [11-13], the rapid growth of eHealth offers a psychological treatment model that can potentially reduce or obviate many of these barriers for postnatal women.

### Internet Interventions for Postnatal Depression

Encouraging results of online intervention for problems including panic disorder, anxiety, posttraumatic stress [14-17], and depression have been reported [14,18,19] and such treatments can have similar efficacy to face-to-face therapy [19-23]. Reviews of the evidence report that although self-guided interventions for depression have benefit to users [14,18,19], even low-intensity guided support from coaches or therapists helps to increase adherence to online mental health treatments [24,25]. Typically, therapeutic effects can be achieved by online interventions that offer low-intensity guidance of less than 3 contact hours in a 6-week program. Guided support can also provide a secondary “safety net” for individuals whose symptoms deteriorate during online treatment.

We developed a PND intervention (MumMoodBooster) with low-intensity guided support based on cognitive behavioral therapy (CBT), which is an established treatment of choice for depressive disorders with its efficacy supported by much research [26,27]. MumMoodBooster was adapted from our Getting Ahead of Postnatal Depression program, which is specifically adapted for the needs of postnatal women (eg, presenting behavioral strategies before cognitive content. Postnatally depressed women, overwhelmed by the demands of infant care, are often not ready to engage in cognitive tasks prior to some behavior change [28]). Previously, we have reported fully on the formative development and systematic usability testing of the MumMoodBooster program [11]. The program developmental process followed an iterative staged approach [29] recommended for development and testing of behavioral interventions [30,31]. We have also demonstrated clinical efficacy in an uncontrolled feasibility trial [13], which showed excellent adherence and acceptability. Of all users, 87% completed all six program sessions; of those women meeting diagnostic criteria for depression at baseline, 90% no longer met criteria after treatment [13].

MumMoodBooster is now one of two evaluated Internet interventions for PND. O’Mahen and colleagues have also developed and tested Netmums, a guided online behavioral activation (BA) treatment [12,32]. MumMoodBooster is a briefer intervention, (6 sessions vs 12 in Netmums) and includes cognitive therapy as well as BA, but both programs include regular telephone support. This is accomplished by a low-intensity, nontherapeutic coaching role in the case of MumMoodBooster and more frequent calls by mental health workers trained in a “high-intensity perinatal-specific BA approach” in the case of Netmums. In the antenatal period, a recently pilot-tested computerized CBT intervention for depression among pregnant women has also shown significant improvements on self-report psychometric measures [33].

Here we report on the efficacy of the MumMoodBooster intervention delivered with low-intensity guided telephone support in a randomized controlled trial (RCT) compared to a treatment as usual (TAU) condition. We included a sample of women diagnosed with a depressive disorder and reassessed diagnostic status 12 weeks after enrollment. We hypothesized that MumMoodBooster would lead to a reduction in depressive symptomatology and an increased rate of remission from the diagnosed depressive episode compared to TAU. Current evidence points to similar potential for efficacy between online and face-to-face CBT for depression in general. Our study sought first to establish whether a PND-specific online CBT program is clinically effective compared to TAU care practices.

## Methods

### Design

This was a parallel 2-group RCT comparing MumMoodBooster to TAU. The main outcomes were remission from the depressive episode and severity of symptoms of depression at 12 weeks postenrollment. See Multimedia Appendix 1 for the study's CONSORT-EHEALTH checklist [68].

### Ethics

The study was approved by the Austin Health Human Research Ethics Committee (approval number H2012/04682) and informed consent was obtained from all participants.

### Recruitment

Recruitment occurred between March 2013 and July 2014. Participants were women resident in Australia aged 18 years and older with a child aged less than 1 year. A stepped process was used to recruit eligible participants: initial screening, clinical assessment, baseline data acquisition, and randomization. The research project was available to women across Australia, in both rural and metropolitan areas. Marketing focused on Internet campaigns using Google AdWords, Facebook, and Twitter. The project was also advertised to Maternal and Child Health Centres in Melbourne who were encouraged to direct clients to the project. Messages prompted interested individuals to obtain more information and to begin a screening process by visiting the secure project recruitment website or to call project staff directly.

### Screening

Screening criteria were determined online as follows: Australian residency, 18 years of age or older, English speaking, less than 1 year postpartum, Internet access with regular email use, an Edinburgh Postnatal Depression Scale (EPDS) [34,35] score of 11 to 23, no current treatment for depression (medication or psychotherapy), and a score of less than 3 on item #10 of the EPDS (indicating frequent thoughts of self-harm).

Individuals who satisfied screening criteria were emailed a participant information and consent form to complete and return by email. Consenting women were subsequently telephoned by a psychologist/psychology trainee to explain the study and to schedule a clinical assessment. At this stage, women who scored 1 to 2 on item #10 of the EPDS were asked a series of questions following the risk assessment protocol of Simon and colleagues [36] to determine intent, lethality, access to means, and history of suicide attempts. Those deemed to be at risk for suicide were excluded and referred to receive immediate crisis attention.

### Clinical Assessment and Inclusion/Exclusion Criteria

Following initial telephone contact, eligible consenting women were assessed by telephone by a clinical psychologist/psychology trainee using the Structured Clinical Interview for *DSM-IV* (SCID-IV) [37,38]. Inclusion criteria based on the SCID-IV assessment were (1) meeting criteria for a major depressive disorder or (2) meeting criteria for a minor depressive disorder. Exclusion criteria were (1) current substance abuse, (2) current and past manic/hypomanic symptoms, (3) posttraumatic stress disorder, (4) alcohol abuse or dependence, (5) depression with psychotic features, (6) risk of suicide as per risk protocol, and (7) current active treatment for depression (medication or psychotherapy). All assessed women were asked to nominate a contact health professional (eg, a general practitioner) to whom the project could send notification of her diagnosis.

### Baseline

Women satisfying all eligibility criteria were told verbally about the unique log-in they could use to complete the baseline (prerandomization) questionnaires.

### Randomization

Women who completed the baseline assessment questionnaires were randomized immediately online to either MumMoodBooster or to TAU. The randomization procedure used a 1:1 allocation ratio and a pregenerated permuted blocks allocation schedule with the sequence concealed from the researchers consistent with CONSORT standards [39]. Treatment allocation to condition was revealed in a phone call. Women allocated to MumMoodBooster were told verbally about how to begin accessing the program. Given the nature of the intervention, participants could not be blinded to treatment beyond the point of allocation.

### Treatment Conditions

#### MumMoodBooster

**Figure 1:**
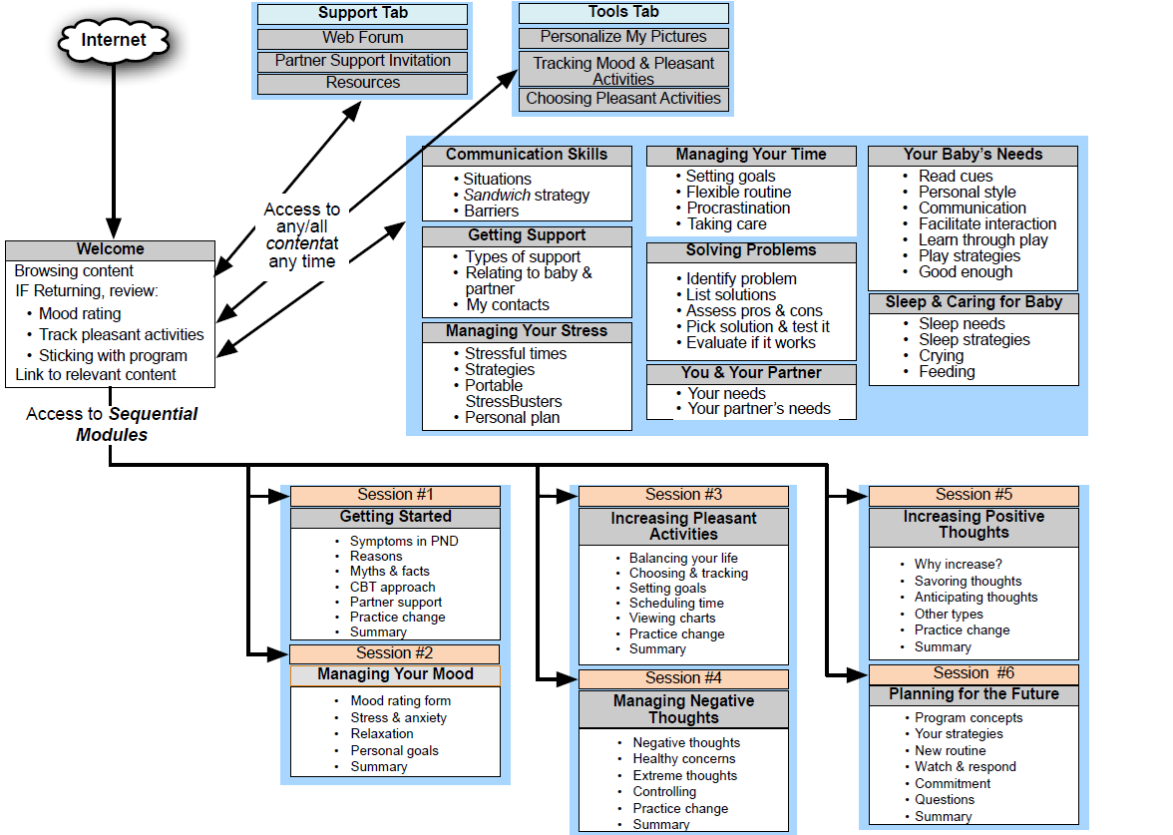
Diagrammatic structure of the MumMoodBooster postnatal depression program.

The structure and content of the MumMoodBooster intervention and its associated websites (partner’s website, coach’s website, administrative website) have been described in detail elsewhere [13]. Figure 1 shows the structure of the MumMoodBooster program.

**Figure 2:**
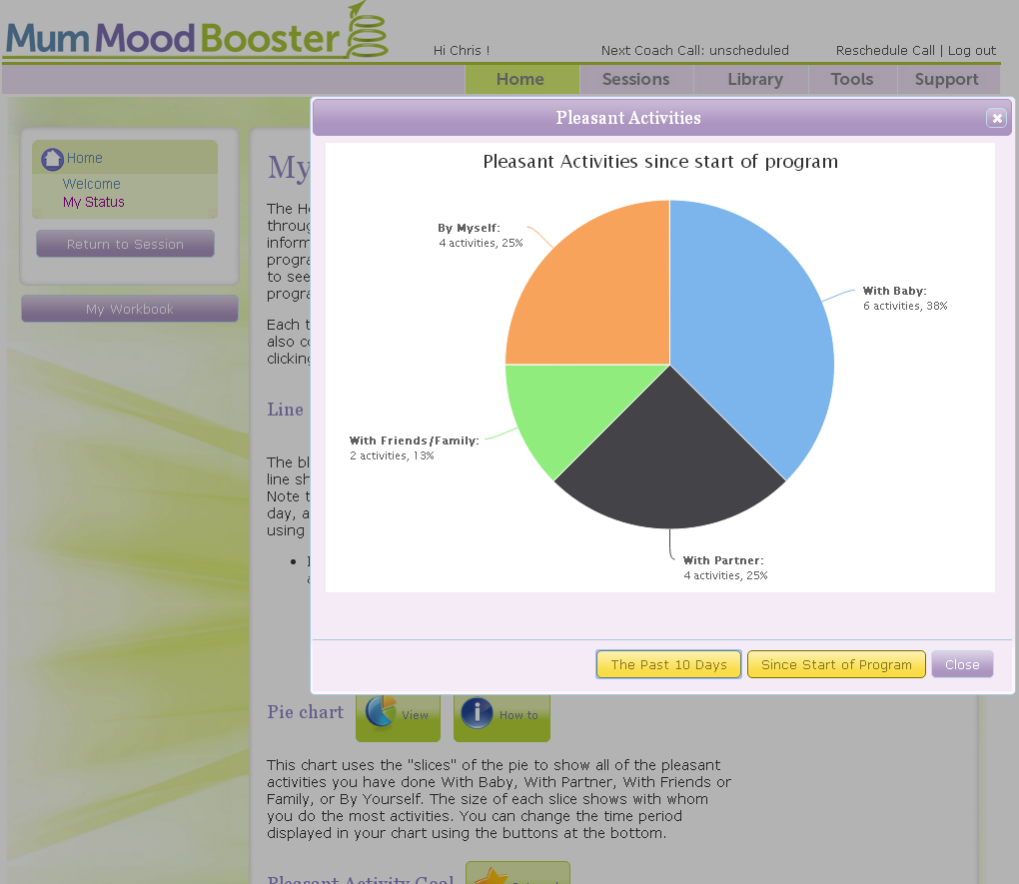
The charting function on a user’s “My Status” page displaying a breakdown of pleasant activities since starting the program.

Treatment consisted of six interactive sessions that were sequentially accessed and designed to encourage optimal engagement and behavior change. For illustration, Multimedia Appendix 2 contains a selection of screenshots from the program. Each session began with an autoplay video introducing session goals and content. Each session presented content using text, animations, video introductions and case vignettes, and audio and video tutorials. The program encouraged participants to personalize their program content, for example by typing in personal lists of pleasant activities, typing in personal goals, and uploading their own photos to be displayed on program webpages. Users could view online and print out a personal workbook summarizing their personalized content. For example, the “my workbook” function gave users feedback on specific strategies that they themselves had identified as helpful together with reminders of how to bring them into play at the earliest warning signs. Self-monitoring tools required participants to enter their own mood and activity data and enabled daily tracking and online charting of both mood and pleasant activities (Figure 2).

Initial steps of the program provided explicit direction, whereas later steps encouraged participants to assume greater responsibility for managing their own plan for change. Later sessions commenced by reviewing previous material before presenting new content and concepts. The program’s charting function was used to help participants see the functional relation of mood and activity levels. Information from past sessions was used to reinforce gains made, to tailor subsequent program content, and to provide ipsative feedback. Although participants were allowed to set their own pace, of particular importance because women with PND are often overwhelmed by the demands of infant care, the program encouraged the completion of all six sessions at a rate of one session per week. A printable summary was used to describe key content covered in each session and provided a tailored list of recommended home practice activities. Participants were also granted unrestricted access to browse additional “library” articles on relevant topics ranging from relaxation, to problem solving, to parenting support resources. Participants also had unrestricted access to a monitored peer-based Web forum on which they could post, read, and comment on messages from other program participants. Additionally, in recognition of the role of women’s partners [40], participants received access to a library article on “You and Your Partner” and were able to send an invitation to their partner to access the related partner support website (separate log-in process for partners) with information on PND.

Participants in MumMoodBooster received guided support from a telephone coach to assist and encourage them in their use and practice of particular strategies (coaches were instructed to spend a maximum of 30 minutes per week per participant). There were 7 coaches (3 graduate psychology trainees, 3 clinical psychologists, and 1 health psychologist) who were supported and supervised by 2 senior psychologists. Training for the coaching role involved working through the program (as if they were a participant), reading the coach manual, observing other coaches’ complete calls, and a verbal explanation from a senior psychologist about the role and the tasks involved. Content of coaching calls adhered to a manualized script with the defined and limited aims of reinforcing progress; encouraging engagement, practice of strategies, and completion of tasks; and introducing the themes of upcoming sessions. The role of the coaches was only to support mothers in using the program and they were instructed to refrain from giving clinical guidance, but rather to point participants to relevant program content that may address their questions (similar to the “technician” role described by Titov and colleagues [41]). Telephone coaches accessed a secure administrative website to view status reports of participant’s program usage to make support and encouragement consistent with progress. Women were also sent automated email prompts as reminders to complete the 12-week online assessment and to encourage visits to the program.

#### Treatment as Usual

Participants in the TAU condition received the same clinical assessment as those in the MumMoodBooster condition. In this condition (as for those in the MumMoodBooster condition), women’s nominated health professional received a written notification of the depressive diagnosis that encouraged them to consult with their patient regarding mental health care needs and to form a collaborative care plan with the patient’s other relevant health care practitioners. In most cases, women nominated their Maternal and Child Health Nurse (MCHN) or general practitioner (GP) who were then free to treat or to refer to other services/agencies as they judged appropriate, as would normally happen where specialized programs are not available. Therefore, in practice, TAU varied at the discretion of each participant’s nominated health professional and was expected to include a heterogeneous mix of interventions/supports. TAU participants were also provided with links to general Internet resources on mental health. They also received the email prompts to complete online assessments.

### Safety Monitoring

Safety monitoring was used to provide crisis intervention and referrals to mental health specialty care if needed and a reminder of the emergency contacts provided at baseline assessment. For women in the MumMoodBooster condition, these were carried out by the allocated telephone coach. For women in TAU, a separate safety monitor was allocated (from the same pool of staff as the telephone coaches). All study participants were monitored via safety calls on five occasions: at baseline and at weeks 3, 5, and 9 (immediately posttreatment) and at 12 weeks postenrollment. We used the protocol of Simon et al [36] to check for depressive symptoms and adverse events. If women scored 5 points above their baseline PHQ-9 score at any time, or if they responded positively to the PHQ-9 item on thoughts of self-harm, then a risk assessment was conducted. This included asking about suicidal thoughts/thoughts of self-harm, frequency of thoughts, triggers, plans, means, lethality, intention, protective factors, and history of suicide attempts/self-harm behavior.

### Measures

Measures were administered via online questionnaire or by telephone. Demographic data (eg, age, parity, education, income) were gathered both during telephone assessment and in the online battery of baseline questionnaires. The key depression measures (described subsequently) are well validated and their psychometric properties are well described in general populations. The performance and psychometric properties of both generic and perinatal-specific depression measures used in the context of perinatal populations have been recently reviewed and were the subject of a full health technology report in 2009 [42,43].

### Primary Depression Outcomes

Before completion of baseline questionnaires and at 12 weeks postenrollment, trained diagnostic interviewers conducted the SCID-IV [38] by telephone to determine a *DSM-IV* diagnosis of major depression or minor depression [44]. Diagnostic interviewers were blinded to treatment allocation at the 12-week time point.

At baseline and at 12 weeks postenrollment, severity of depressive symptoms was measured online using the revised Beck Depression Inventory (BDI-II) [45]. The BDI-II is a widely used, well-validated, 21-item clinical instrument that measures cognitive, affective, and physiological factors to assess severity of depression. The BDI-II has been used in many studies of perinatal depression [46-50] and has been validated against gold-standard diagnostic criteria in perinatal populations [47].

### Secondary Outcomes

#### Depressive Symptom Trajectory

Participants completed the 9-item PHQ-9 [51,52] at enrollment and at weeks 3, 5, 9, and 12 postenrollment. The PHQ-9 was administered over the telephone during the routinely scheduled safety monitoring calls.

#### Anxiety and Stress Symptom Severity

Participants’ anxiety and stress symptom severity were measured at baseline and at weeks 9 and 12 using the anxiety and stress scales of the Depression, Anxiety and Stress Scales—Short Form (DASS-21) [53]. The DASS-21 is a 21-item, 4-point Likert-type scale. The anxiety and stress scales each have a maximum score of 42 [53]. The DASS manual [53] provides recommended cut-offs for rating the severity of scores as normal, mild, moderate, severe, or extremely severe.

#### Negative Thinking

At baseline and at 12 weeks postenrollment, participants were asked to indicate how frequently over the previous week they had negative thoughts using the 30-item Automatic Thoughts Questionnaire (ATQ) [54]. The ATQ asks respondents to rate their agreement with a series of statements (eg, “My life is a mess”) using a scale from 1 to 5 (1=not at all; 5=all of the time). Maximum score is 150.

#### Behavioral Activation

To measure changes in behavior patterns, at baseline and at 12 weeks postenrollment, women completed the 25-item Behavioral Activation for Depression Scale (BADS) [55]. Respondents rate their agreement with a series of statements (eg, “I stayed in bed for too long even though I had things to do”) on a scale from 0 to 6 with a maximum total score for the scale of 150.

#### Relationship With Partner

Women’s relationships with their partners were assessed using the abbreviated 7-item version of the Dyadic Adjustment Scale (DAS-7) [56] at baseline and at 12 weeks postenrollment. The general satisfaction score was calculated as the sum of all items. Maximum score is 36.

#### Self-Efficacy in Parenting Role

At baseline and at 12 weeks postenrollment, we used the Parenting Sense of Competence Scale (PSOC) [57] that asks respondents to rate the extent of their agreement with 7 items designed to assess self-perception of knowledge and competence in the mothering role. Statements (eg, “I honestly believe I have all the skills necessary to be a good mother to my baby”) are rated from 1 to 6 (1=strongly disagree; 6=strongly agree). Maximum score is 42.

#### Engagement in MumMoodBooster Program

Website analytic tools and database flags allowed us to measure MumMoodBooster program usage in an unobtrusive manner, generating records of number and duration of visits to the website and number of program sessions attended.

#### Treatment Satisfaction and Helpfulness

Satisfaction with the MumMoodBooster program was assessed using a 4-point Likert scale (1=not at all satisfied; 4=very satisfied). Helpfulness of phone coach calls was similarly assessed using a 4-point Likert scale (1=not at all helpful; 4=very helpful).

#### Use of Other Supports/Treatments

Participants were also asked to provide details of access to other support services and treatments during the study interval.

### Statistical Analysis

The primary outcomes were changes in depressive diagnostic status (assessed by the SCID-IV) and depression symptom severity (BDI-II). The categorical outcome (ie, diagnostic status) was analyzed using a contingency table and chi-square test. The continuous outcome (ie, BDI-II) was analyzed in general linear models, which accounted for baseline values as a covariate. Continuous repeated measures data (PHQ-9) were analyzed in mixed-effects growth models using restricted maximum likelihood. Log-likelihood ratio and Akaike information criterion were used to assess model fit.

Consistent with CONSORT standards [58,59], all primary analyses involved planned contrasts of the MumMoodBooster condition versus the TAU control condition with all randomized participants analyzed in their allocated treatment condition. For anxiety and stress symptom severity (DASS-21 Anxiety scale and DASS-21 Stress scale), because transformation failed to improve normality, the nonparametric Mann-Whitney *U* test was used for between-group comparison. Effect sizes were expressed as Cohen’s *d* [60] with 95% confidence intervals.

There was some missing data (<5%) on the primary outcomes. Little’s missing completely at random (MCAR) test [61] revealed that these data were missing completely at random (χ^2^_335_^=^303.56, *P=*.89). Given the minimal and random nature of missing data, the highly conservative “worst-case” imputation method [41] was used for intention-to-treat analysis. This method replaces missing outcome values in the intervention and control conditions with the least favorable and most favorable scores observed within those respective conditions [41], thus providing a sensitivity analysis of the robustness of the observed treatment effect to type I error [62]. Results are presented on the basis of the intention-to-treat analyses. Computations were executed using IBM SPSS Statistics version 22 (IBM Corp, Armonk, NY, USA).

### Power and Sample Size

For the outcome measure of depressive symptom severity (BDI-II), data from a previous trial of CBT [28] for PND provided relevant estimates of variability in baseline scores (mean 23, SD 8.1 points). On this basis, a difference of 6.5 points would be necessary to move mean scores from the “moderately severe” to the “minimal” category of depressive symptoms specified by Beck and colleagues [63] (cut-off between these categories=17 points). We considered this to be the minimum clinically important difference in continuous scores of depression severity [28]. With a power of 0.8 at α=.05, the required n=15.7(8.09/6.5)^2^=24.3, which rounds to 25 per group. Thus, we aimed for a total sample size of N=50 to achieve sufficient power to detect a clinically important difference in the primary measure of depressive symptom severity.

## Results

### Sample

**Figure 3:**
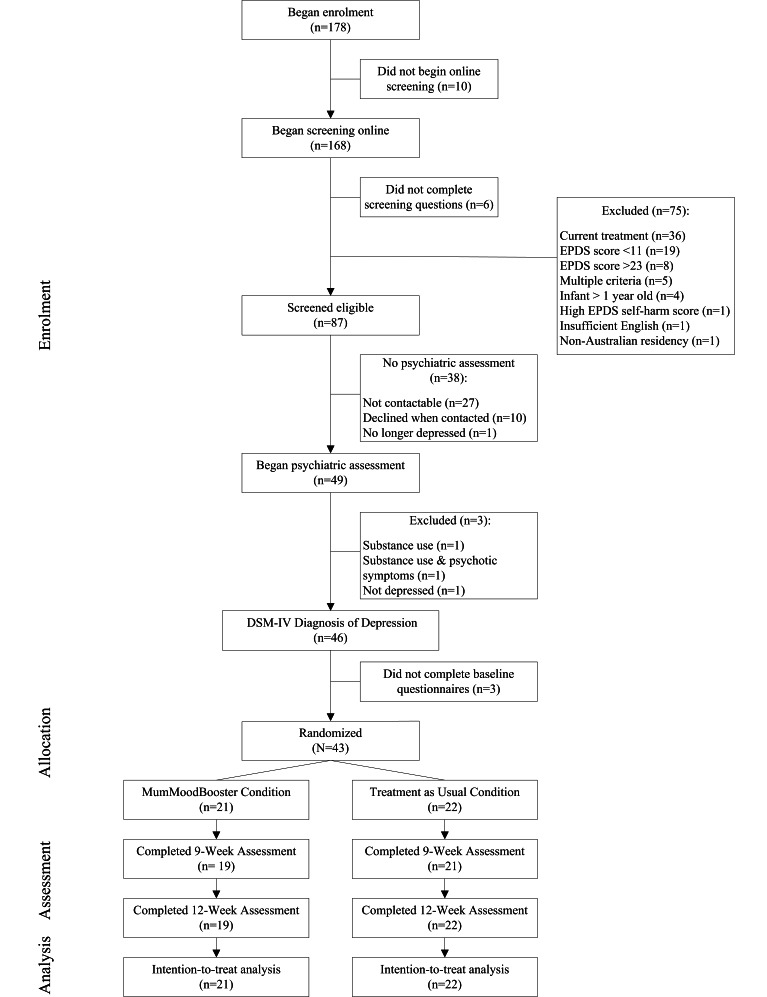
CONSORT diagram of participant flow through the study.

A total of 178 women began the study registration process (see Figure 3). By the end of the recruitment period, 43 mothers were randomized (24.2% of registrants) to either the MumMoodBooster condition (n=21) or the TAU condition (n=22). Figure 3 details the reasons for attrition between registration and randomization. Twelve weeks following enrollment, two women in the MumMoodBooster condition failed to complete online assessment questionnaires and telephone diagnostic interviews (all women in TAU completed the 12-week assessments).

Women in the two conditions appeared comparable in terms of baseline characteristics (Table 1). As recommended by the CONSORT Statement [58], no significance tests of between-group differences were conducted on baseline data.

**Table 1:** Baseline characteristics of participants (N=43).

Characteristic		MumMoodBooster (n=21)	TAU (n=22)
Mother’s age (years), mean (SD)		31.7 (4.6)	31.5 (4.3)
Baby’s age (months), mean (SD)		6.52 (2.8)	6.15 (3.1)
EPDS at screening, mean (SD)		16.6 (3.1)	15.8 (2.8)
Born in Australia, n (%)		18 (86)	21 (96)
Current major depression, n (%)		20 (95)	20 (91)
Current minor depression, n (%)		1 (5)	2 (9)
Past major depression, n (%)		14 (67)	15 (68)
**Relationship status, n (%)**			
	Married/Living with partner	18 (86)	20 (91)
	Single	3 (14)	2 (9)
**Education, n (%)**			
	Did not finish school	0 (0.0)	1 (5)
	High school only	2 (9)	3 (14)
	Certificate level	4 (19)	3 (14)
	Diploma level	5 (24)	4 (18)
	Undergraduate degree	6 (29)	7 (32)
	Postgraduate degree	4 (19)	4 (18)
**Number of children (including most recent baby), n (%)**			
	1	7 (33)	7 (32)
	2	7 (33)	11 (50)
	≥3	7 (33)	4 (18)
**Family income (AUS$), n (%)**			
	≤$20,000	0 (0)	1 (5)
	$20,001-$40,000	0 (0)	1 (5)
	$40,001-$60,000	2 (10)	1 (5)
	$60,001-$80,000	3 (14)	6 (27)
	>$80,000	13 (62)	13 (59)
	Not divulged	3 (14)	0 (0)

In the MumMoodBooster condition, 20 of 21 women (95%) were diagnosed with current major depression and 1 of 21 women (5%) with minor depression. In the TAU condition, 20 of 22 women (91%) met diagnostic criteria for current major depression and 2 of 22 women (9%) for minor depression (Table 1).

The mean age of mothers was close to the most recently available Australian national average (30 years [64]) and their infants averaged just over 6 months of age. The sample contained a higher proportion of women born in Australia than the latest national average (national average=70%) and a lower proportion of first-time mothers (national average=43%). The mean EPDS at screening was 16.2 (SD 2.9), similar to the mean observed in other Australian samples of depressed perinatal women [47]. As reported in studies of major risk factors for PND [65], 67.4% (29/43) of the cohort had experienced a past major depressive episode.

### Primary Depression Outcomes

**Figure 4:**
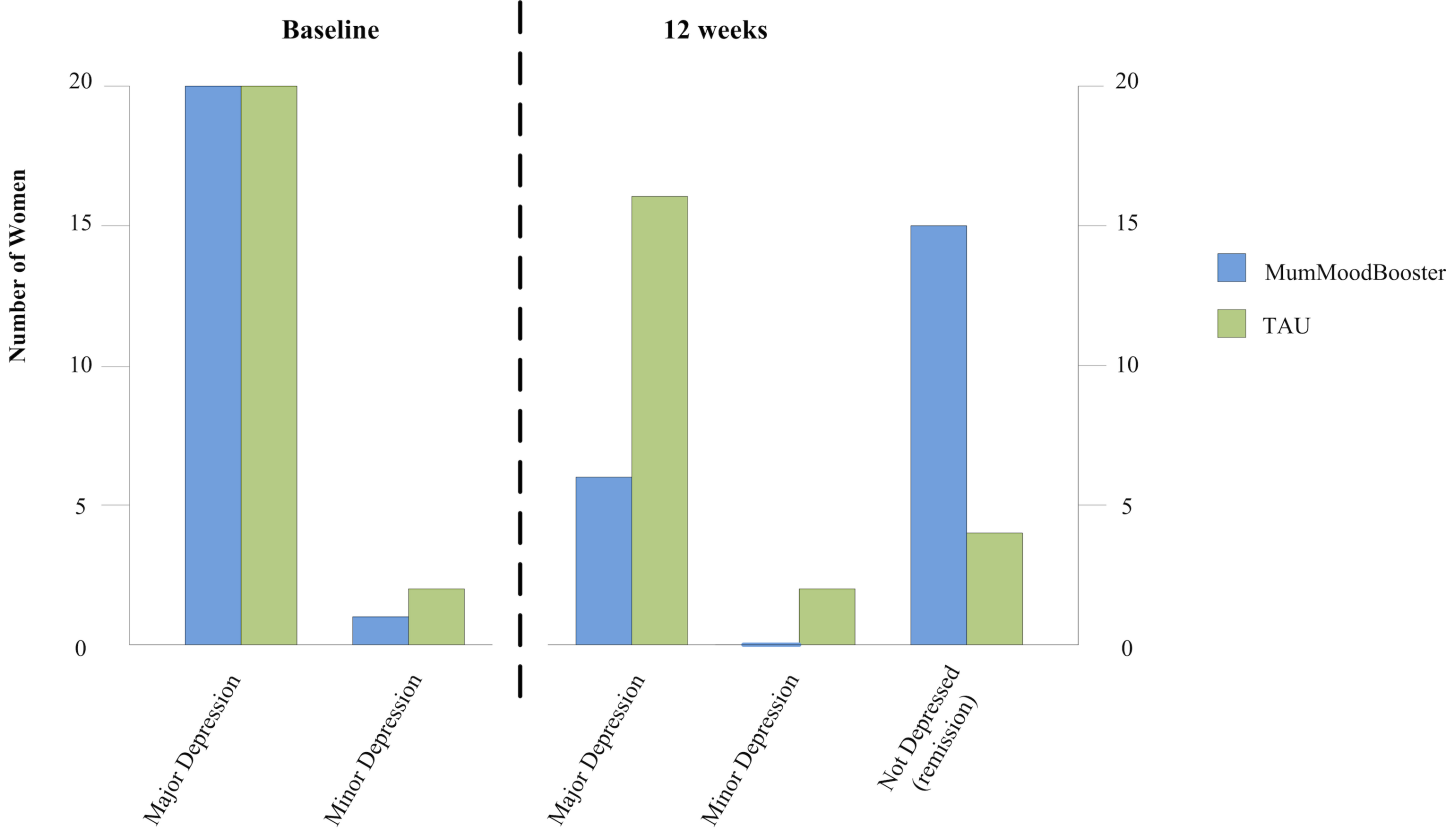
DSM-IV diagnoses at baseline and 12 weeks postenrollment.

In the MumMoodBooster condition, 79% (15/19) of women no longer met *DSM-IV* criteria for depression at 12 weeks; this was 18% (4/22) in the TAU condition. In the intention-to-treat analysis, a chi-square test revealed a statistically significant between-group difference in the frequency of depressive diagnosis (major or minor depression) at 12-week follow-up (Yates corrected χ^2^_1_=10.3, *P*=.001). Figure 4 shows the proportions on which the intention-to-treat analysis was conducted.

Similarly, women in the MumMoodBooster condition also showed reduced severity of depression symptoms, whereas those in the TAU condition displayed little improvement (see Table 2). After controlling for baseline scores, mean depression symptoms on the BDI-II in the intervention group at 12 weeks were significantly lower than that of the TAU group (*P=*.01) and this represented a large effect size (Table 2). Results for both diagnostic status and BDI-II scores were also statistically significant in observed-case analyses before imputation.

**Table 2:** Severity of depression, anxiety, and stress symptoms over time.^a^

Outcome measure^b^		MumMoodBooster, mean (SD) n=21	TAU, mean (SD) n=22	*F*_1,40_	*U*	*P*	*d* (95% CI)
**BDI-II**							
	Baseline	25.3 (6.4)	26.3 (8.6)				
	12 weeks	14.5 (12.2)	23.0 (7.5)	7.4		.01	0.83 (0.20, 1.45)
**DASS Anxiety**							
	Baseline	9.0 (7.0)	6.7 (5.3)				
	9 weeks	5.0 (6.2)	5.9 (4.3)		176.0	.17	0.18 (–0.42, 0.78)
	12 weeks	4.2 (5.5)	5.4 (3.0)		153.0	.05	0.27 (–0.33, 0.87)
**DASS Stress**							
	Baseline	22.1 (8.0)	20.7 (7.2)				
	9 weeks	14.2 (9.0)	18.5 (7.1)		142.5	.03	0.54 (–0.07, 1.15)
	12 weeks	13.1 (8.7)	18.1 (10.2)		153.5	.06	0.53 (–0.08, 1.14)

^a^ Tabled values are from intention-to-treat analyses after imputation of missing values.

^b^ BDI-II: Beck Depression Inventory II; DASS: Depression, Anxiety and Stress Scales.

### Secondary Outcomes

#### Trajectory of Depressive Symptoms

**Figure 5:**
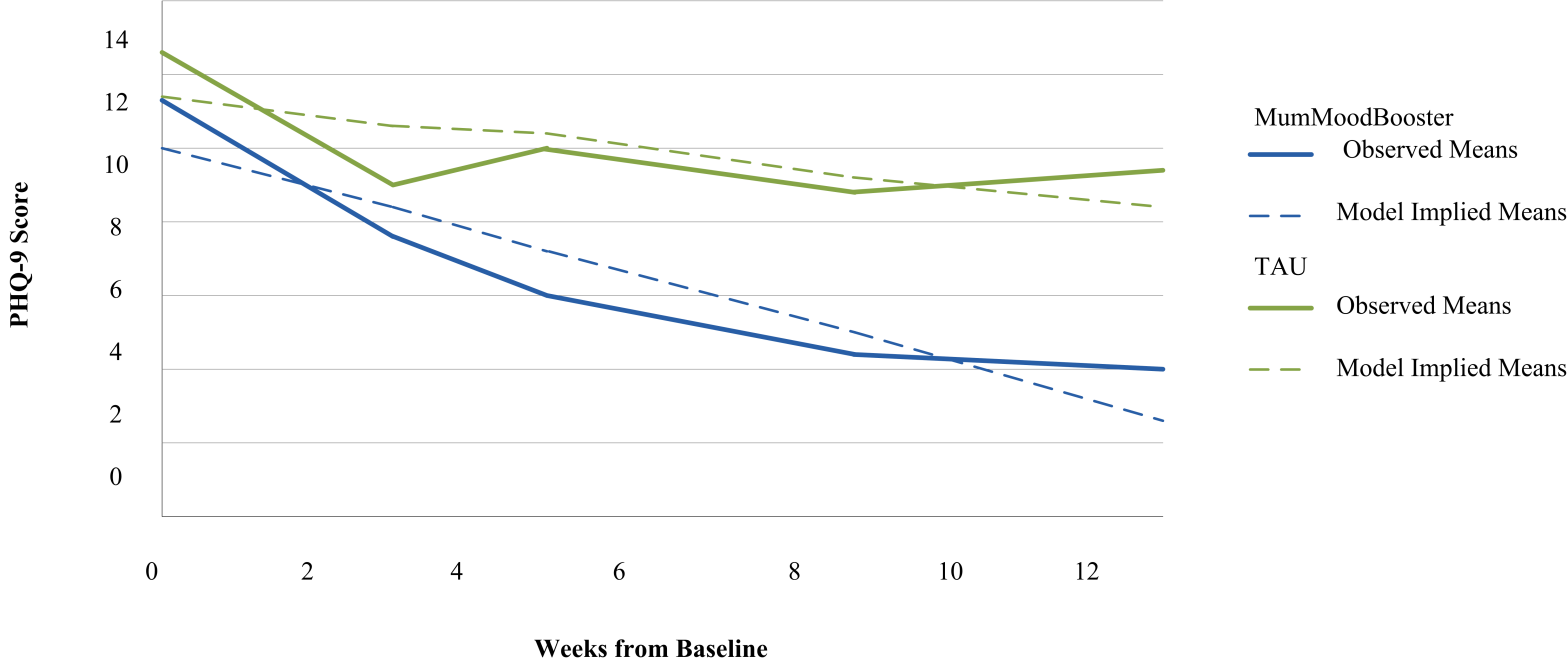
Longitudinal trajectories of Patient Health Questionnaire-9 (PHQ-9) scores from baseline to 12 weeks.

Figure 5 shows PHQ-9 scores across the study period for both conditions. There was no significant between-condition difference in PHQ-9 scores at baseline. A substantial reduction in PHQ-9 scores occurred from enrollment to 12 weeks in the MumMoodBooster condition (mean 7.2 point drop). However, for the TAU condition, PHQ-9 scores fluctuated slightly with only a small reduction over the same time period (mean 3.3 point drop). A mixed-effects growth model (random intercept model with a linear trajectory) revealed a significant linear decrease in PHQ-9 values for all study participants (estimate=–0.23, SE 0.08, *P*=.01, partial *r* =–.22) and a differential trajectory between conditions, with MumMoodBooster participants’ scores decreasing (improving) at a greater rate (estimate=–0.34, SE 0.12, *P*=.01, partial *r*=–.23).

#### Anxiety and Stress Symptom Severity

At baseline, mean anxiety scores were within the normal-to-mild range of severity (0-9). Mean baseline stress scores fell in the moderate range of severity (19-25). At 9 weeks (immediately posttreatment) and at 12 weeks postenrollment, average symptom severity on the Anxiety scale of the DASS-21 was not significantly lower in the MumMoodBooster condition compared to the TAU condition (Table 2) and represented small effect sizes at both times (*d*=0.18 and *d*=0.27, respectively; see Table 2). Mean symptom severity on the Stress scale of the DASS-21 showed a significant between-group difference favoring the MumMoodBooster condition at 9 weeks, but not at 12 weeks. These differences represented medium effect sizes at both times (see Table 2; *d*=0.54 and *d*=0.53, respectively).

#### Negative Thinking and Behavioral Activation

Mean scores for measures of both negative thinking (ATQ) and behavioral activation (BADS) showed some improvement in both the MumMoodBooster and TAU conditions (see Table 3), with medium to large effect sizes favoring the MumMoodBooster condition at 12 weeks postenrollment.

**Table 3:** Secondary outcomes at baseline and 12 weeks.^a^

Outcome measure^b^		MumMoodBooster, mean (SD) *n*=21	TAU, mean (SD) *n*=22	*F*_1,40_	*P*	*d* (95% CI)
**ATQ** ^c^						
	Baseline	70.19 (16.49)	80.55 (22.89)			
	12 weeks	56.33 (26.73)	72.05 (20.78)	3.71	.06	0.61 (–0.01, 1.22)
**BADS** ^d^						
	Baseline	79.86 (15.76)	78.27 (15.08)			
	12 weeks	103.24 (24.49)	84.77 (17.07)	7.96	.01	0.86 (0.23, 1.48)
**DAS-7** ^d^						
	Baseline	21.10 (6.95)	19.41 (7.92)			
	12 weeks	22.67 (6.43)	21.23 (6.36)	0.10	.75	0.10 (–0.50, 0.70)
**PSOC** ^d^						
	Baseline	27.52 (7.50)	21.23 (7.37)			
	12 weeks	30.19 (7.53)	23.36 (7.85)	2.54	.12	0.53 (–0.08, 1.13)

^a^ Tabled values are from intention-to-treat analyses after imputation of missing values.

^b^ ATQ: Automatic Thoughts Questionnaire; BADS: Behavioral Activation for Depression Scale; DAS-7: Dyadic Adjustment Scale: PSOC: Parenting Sense of Competence.

^c^ Lower score is superior.

^d^ Higher score is superior.

#### Relationship With Partner and Parenting Self-Efficacy

The intervention had no statistically significant effect on partner relationships (DAS-7 scores), although its impact on the parenting self-efficacy measure (PSOC) constituted a medium effect size (Table 3).

#### Engagement in MumMoodBooster Program

All women offered the MumMoodBooster intervention (21/21) completed four or more sessions and 86% (18/21) completed all six sessions. Women visited the program on a mean of 20.5 occasions (SD 10.6) and the mean number of sessions attended was 5.7 (SD 0.7). The total time spent using the online program averaged 370 minutes (range 120-1076). Content on the Web forum was viewed, to varying degrees, by all participants and 57% (12/21) posted their own comments to the forum. The mean number of library articles accessed was 4 out of a possible 8. Of the six scheduled coach calls, participants completed a mean 4.3 calls (SD 2.2, range 0-6). A total of 76% (16/21) of participant partners accessed the partner support website.

#### Treatment Satisfaction and Helpfulness

Of the 21 women in the MumMoodBooster condition, 90% (19/21) provided ratings of the program’s satisfaction and the helpfulness of coaching calls. Mean satisfaction ratings were in the moderately satisfied range (mean 3.1, SD 0.60, range 2-4) on a 4-point scale. Similarly, mean ratings of the helpfulness of telephone coach calls were in the moderately helpful range (mean 3.2, SD 0.89, range 1-4) on a 4-point scale.

#### Use of Other Supports/Treatments

**Figure 6:**
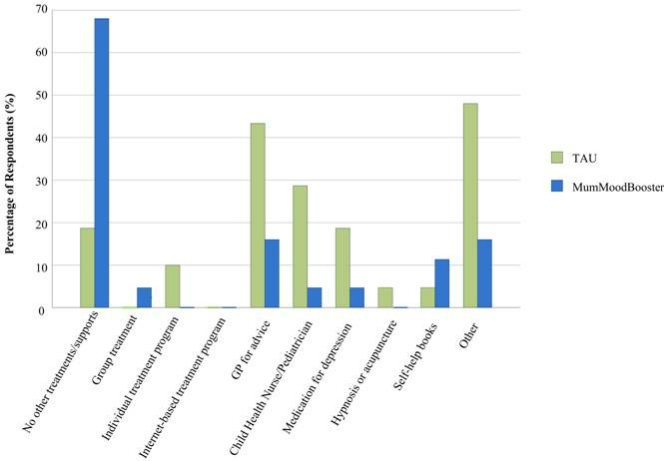
Other supports and treatments accessed during the trial (n=6 women in the MumMoodBooster condition and n=17 in the TAU condition reported accessing other supports).

At the 12-week assessment, all study participants were asked: “Since you enrolled in the study, which of the following products or programs have you used to manage your mood? (choose all that apply).” A checklist was presented and participants checked the items relevant to their usage. The possible items were (1) I participated in a group treatment program, (2) I participated in an individual treatment program, (3) I participated in another Internet-based treatment program, (4) I saw my doctor who gave me advice, (5) I saw my Maternal & Child Health Nurse/Pediatrician who gave me advice, (6) I took medication for depression, (7) I used hypnosis or acupuncture, (8) I read self-help books, and (9) other (please specify). Of the respondents in the MumMoodBooster condition, 32% (6/19) reported that they had accessed one or more sources of support, whereas 81% (17/21) of those in TAU reported using one or more supports while enrolled in the trial; this was a statistically significant difference (continuity corrected χ^2^_1_ *=*10.3, *P*=.002). Figure 6 shows the types of supports accessed in both groups.

Of note, individual psychological treatment was reported only by TAU participants, whereas one respondent (5%) in the MumMoodBooster condition reported commencing the use of antidepressant medication compared to 4 (19%) in TAU. More respondents in the TAU group reported having accessed their GP, child health nurse, or pediatrician. Various supports reported by participants, categorized collectively as “other,” included Internet research, meditation, telephone helplines, and talking to friends/other mothers. More respondents in the TAU group (10/21, 48%) accessed these “other” supports compared to the MumMoodBooster condition (3/19, 16%).

## Discussion

Postnatal depression is prevalent, with enormous negative effects on maternal and infant well-being, yet postnatally depressed women receive adequate treatment in less than 10% of cases [66]. Internet interventions offer promise in overcoming some of the main barriers to treatment access and allow women more direct control over their own treatment schedule. By mitigating the difficulty of attending a clinic with a young infant and by reducing barriers such as perceived stigma, these treatments hold the potential to empower depressed perinatal women to take effective steps to overcome their emotional health difficulties.

This RCT demonstrates that the MumMoodBooster intervention results in very good participant engagement with the program, increased rates of remission from diagnosed depression, as well as a significantly more rapid reduction in severity of depressive symptoms. This improvement was evidenced in a sample with baseline depression severity in the moderately severe range and with the vast majority of participants diagnosed with major depression. The intervention produced a 4-fold improvement in the rate of depression remission compared to the TAU condition—even when worst-case results were imputed for the few missing data values.

These results are an encouraging replication of the findings of our previous uncontrolled feasibility study of MumMoodBooster with postnatally depressed women in both Australia and the United States [13]. For example, the observed trajectories of depressive symptoms measured on the PHQ-9 are closely similar in both shape and values, showing rapid initial gains followed by decelerating but continued improvement from around the fifth or sixth week of treatment. Further, treatment adherence, as reflected in the mean number of website visits and CBT sessions viewed, were almost identical in the two studies. Again, similar to the feasibility results, the current RCT found that improvements in automatic thinking and behavioral activation represented medium to large treatment effects favoring the MumMoodBooster intervention, a result in accordance with theoretical expectations regarding the mechanism of action associated with CBT. In both this trial and our earlier feasibility study, we found no evidence of any improvement in women’s relationships with their partners over the study period (as measured by the DAS-7).

This RCT also evaluated changes in symptoms of anxiety and stress, commonly comorbid with depression, and found medium effects favoring the intervention in terms of stress but not anxiety. In interpreting this result, it is worth noting that, at baseline, the mean DASS-21 Anxiety score for participants already fell inside the normal range for anxious symptoms. Although comorbid anxiety symptoms are commonly reported among women with PND, in this sample there may have been little potential gain to make in terms of reducing anxiety scores.

The study has some limitations. First, the RCT was based on a relatively small sample size meaning that some caution is required in trying to generalize our results to the wider perinatal population. Second, women allocated to TAU reported high levels of alternative help seeking and this may have made the detection of true treatment effects relative to TAU more difficult. Conceivably, the comprehensive psychological assessment process for trial inclusion (which is not a consistent feature of TAU in real-world practice) may itself have affected rates of alternative help seeking. It was not unexpected to see the considerable variation in treatments and supports accessed in TAU, as has been discussed in a recent review of what constitutes TAU in the context of RCTs of psychological interventions [67]. However, the possible influence of access to alternative supports and treatments in both groups should be borne in mind when interpreting our results. Last, the follow-up period was short (12 weeks postenrollment) and precluded assessment of the endurance of the treatment effect beyond this time.

Despite these limitations, the robustness of the intention-to-treat analysis tends to uphold the reliability of the results for the primary outcomes. Finally, a major strength of the study is that the efficacy of the MumMoodBooster intervention in treating PND was evaluated against *DSM-IV* diagnostic criteria. This is the first such result using a diagnostic outcome measure in this field of research.

In summary, this RCT confirms and adds to existing evidence for the efficacy of online treatment for PND [12,13]. Apparent strengths of the MumMoodBooster program are its observed ability to rapidly improve symptoms in women with severe diagnosed depression and that it appears highly acceptable. It also appears to help change underlying negative cognitions as well as create behavioral change. As with the study by O’Mahen and colleagues [12], the version of MumMoodBooster evaluated in this RCT included telephone support, but to a lesser degree, and women found this coaching component to be helpful. Online psychological treatment may be particularly relevant for women with PND. It can be accessed in a flexible manner that may better fit a woman’s own time-management needs in caring for her infant. It can be delivered with greater privacy, which may help to address the reluctance of many women to seek traditional forms of treatment due to perceived stigma [8]. Future research might focus profitably on quantifying the value of guided support in such online perinatal interventions and on the potential efficacy of less guided versions or of purely self-guided online treatments for PND. Finally, efficacy relative to traditional, in-person clinical psychological treatment remains to be established. We are currently engaged in a large (n*=*210) randomized trial in Australia designed to directly compare MumMoodBooster with best-practice specialized face-to-face CBT treatment for PND and with an extended period of follow-up (trial registration number: ACTRN12613000881730).

## Multimedia Appendix 1

CONSORT-EHEALTH checklist V1.6.2 [68].

## Multimedia Appendix 2

Selected Screenshots.

## Abbreviations

ATQ: Automatic Thoughts Questionnaire
BA: behavioral activation
BADS: Behavioral Activation for Depression Scale
BDI-II: Beck Depression Inventory
CBT: cognitive behavioral therapy
DAS-7: Dyadic Adjustment Scale
DASS-21: Depression, Anxiety and Stress Scales—Short Form
EPDS: Edinburgh Postnatal Depression Scale
GP: general practitioner
MCHN: Maternal and Child Health Nurse
PHQ-9: Patient Health Questionnaire
PND: postnatal depression
PSOC: Parenting Sense of Competence
RCT: randomized controlled trial
TAU: treatment as usual
