# Supplementary material for: Internet Cognitive Behavioral Therapy for Women With Postnatal Depression: A Randomized Controlled Trial of MumMoodBooster
Source: J Med Internet Res. 2016 Mar 7;18(3):e54. doi: 10.2196/jmir.4993 (PMC4802107; doi:10.2196/jmir.4993)
Supplement: Supplementary file 2 [file jmir_v18i3e54_app2.pdf]

MumMoodBooster - Windows Internet Explorer

https://www.mummoodbooster.com/home/index.jsp

File Edit View Favorites Tools Help

Favorites | Best of the Web | Channel Guide | Customize Links | Free Hotmail | Internet Explorer News | Internet Start | ninemsn | ninemsn: CelebrityFIX... | ninemsn: Money | ninemsn Most popular

MumMoodBooster

Mum Mood Booster

Hi qwertyau! Next Coach Call: Tue 27 Sep 7 PM Reschedule Call | Log out

Home | Sessions | Library | Tools | Support

Home
- Welcome
- My Status

Go to Session 1

My Workbook

Home: Welcome

Welcome to the MumMoodBooster program!

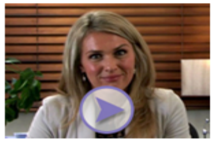

You are about to begin the MumMoodBooster program designed for women who are experiencing postnatal depression. It is based on years of clinical experience and controlled research with hundreds of women with postnatal depression. The goal is to reduce moderate to severe symptoms of postnatal depression.

The program will give you tools that can help you identify both positive and challenging situations in order to take control of your feelings, mood and thinking. It aims to improve the quality and enjoyment you experience in your life. It gives you the chance to learn strategies that can reduce how often you feel depressed in the future, and you can learn how to shorten the time that your depression lasts.

It takes time

There are lots of good reasons to make spending time on this program one of your priorities. It can:

- Be practical for most women to use
- Help you manage your thoughts so that you will feel better
- Improve the quality of your life and relationships
- Help you make long-lasting improvements that become part of your normal routine

It's *Your* program

This program is divided into six sessions, each of which takes about a week to complete. The skills and strategies you learn build on each other. There are lists, plans, and forms that you can customize with your information – which is private so that nobody outside of you and the research team can see it.

Done | Internet | 100%

Start | Removable Disk (E:) | Inbox - Microsoft Outlook | Personal coach training... | MumMoodBooster - ... | How to Do a Screen Ca... | 3:59

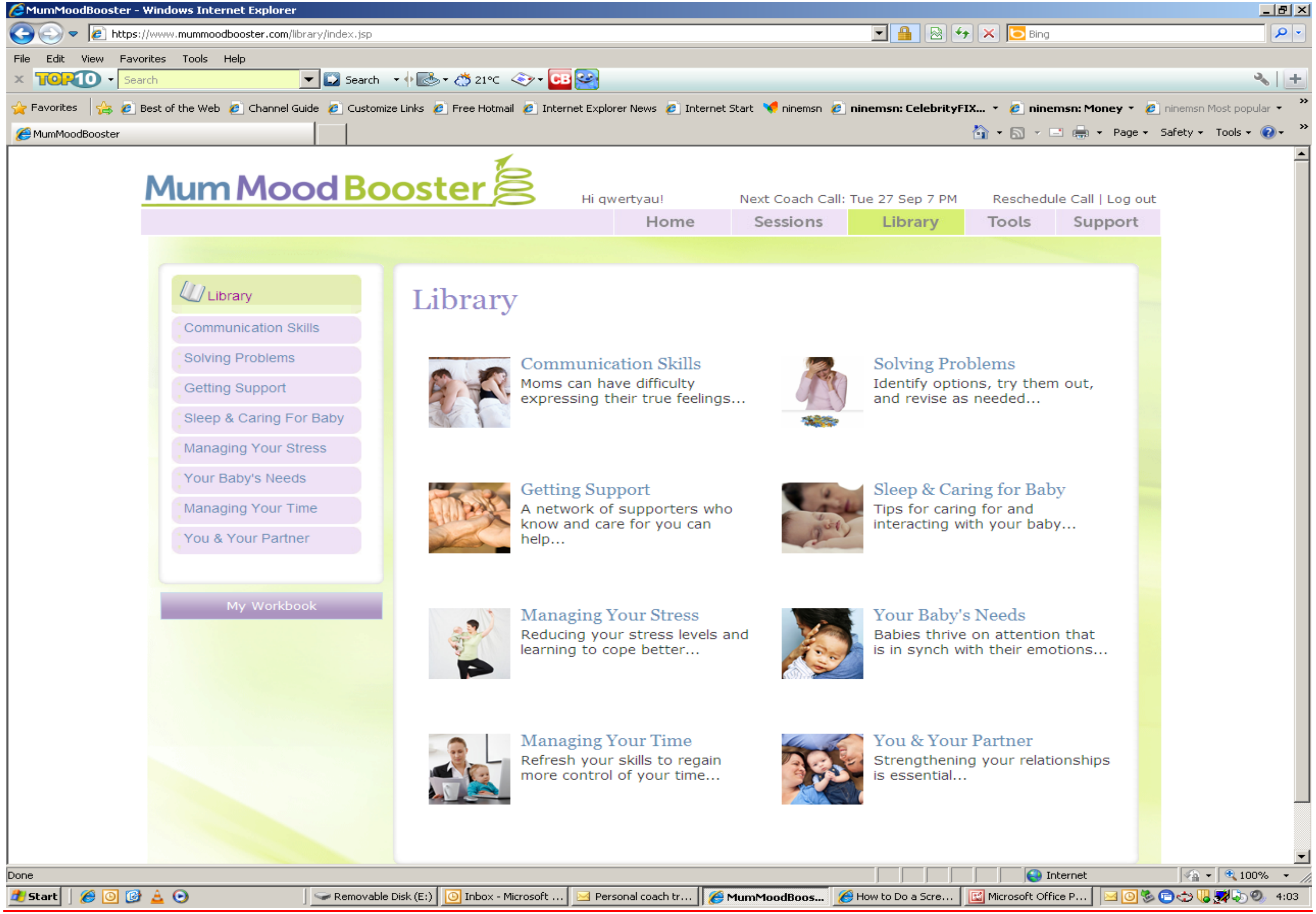
MumMoodBooster - Windows Internet Explorer
https://www.mummoodbooster.com/library/index.jsp
Mum Mood Booster
Hi qwertyau!
Next Coach Call: Tue 27 Sep 7 PM
Reschedule Call | Log out
Home
Sessions
Library
Tools
Support
Library
Communication Skills
Solving Problems
Getting Support
Sleep & Caring For Baby
Managing Your Stress
Your Baby's Needs
Managing Your Time
You & Your Partner
My Workbook
Library
Communication Skills
Moms can have difficulty expressing their true feelings...
Solving Problems
Identify options, try them out, and revise as needed...
Getting Support
A network of supporters who know and care for you can help...
Sleep & Caring for Baby
Tips for caring for and interacting with your baby...
Managing Your Stress
Reducing your stress levels and learning to cope better...
Your Baby's Needs
Babies thrive on attention that is in synch with their emotions...
Managing Your Time
Refresh your skills to regain more control of your time...
You & Your Partner
Strengthening your relationships is essential...
Done
Internet
100%
4:03

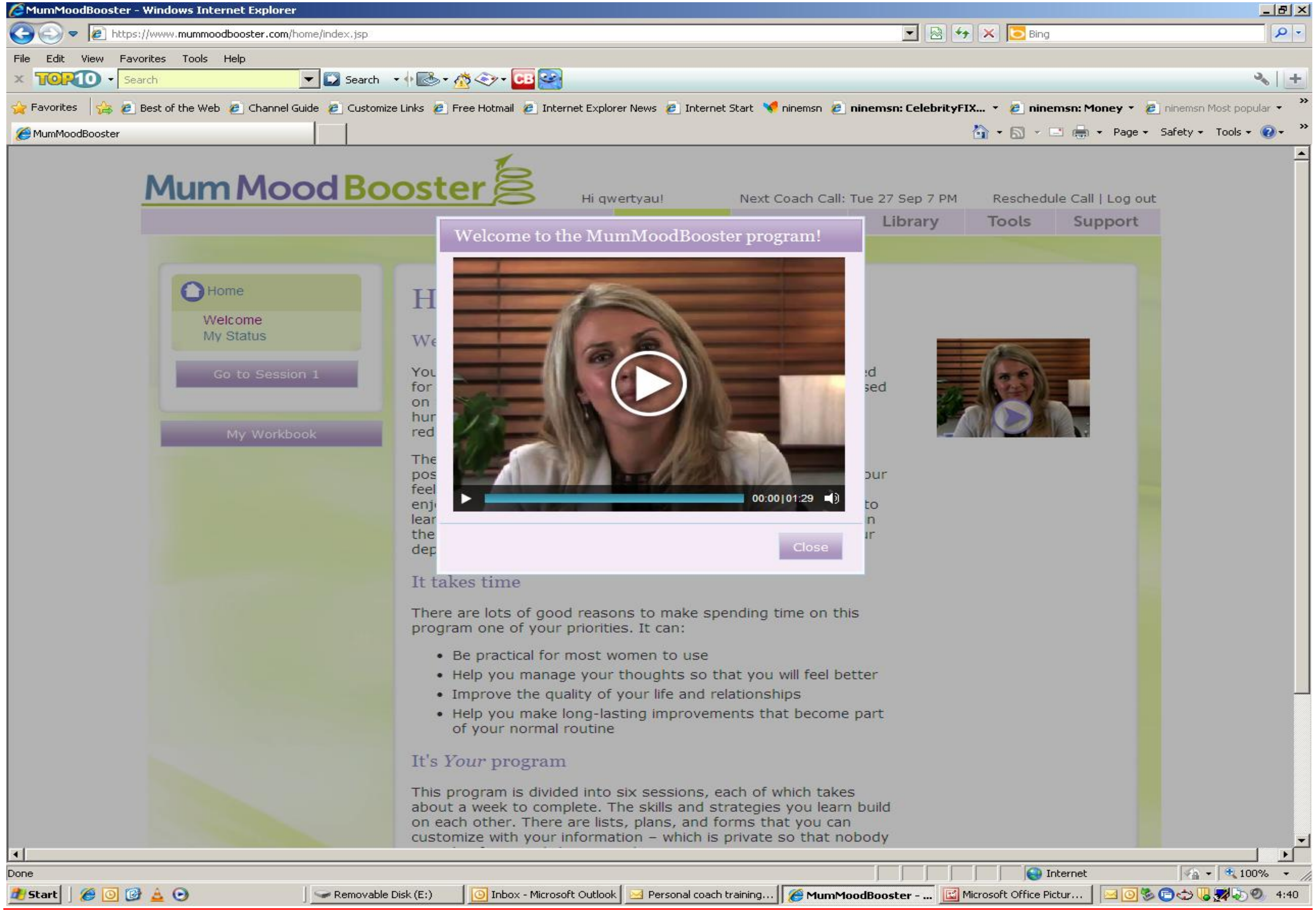
MumMoodBooster - Windows Internet Explorer
https://www.mummoodbooster.com/home/index.jsp
Mum Mood Booster
Hi qwertyau!
Next Coach Call: Tue 27 Sep 7 PM
Reschedule Call | Log out
Library
Tools
Support
Welcome to the MumMoodBooster program!
00:00 | 01:29
Close
Home
Welcome
My Status
Go to Session 1
My Workbook
It takes time
There are lots of good reasons to make spending time on this program one of your priorities. It can:
Be practical for most women to use
Help you manage your thoughts so that you will feel better
Improve the quality of your life and relationships
Help you make long-lasting improvements that become part of your normal routine
It's Your program
This program is divided into six sessions, each of which takes about a week to complete. The skills and strategies you learn build on each other. There are lists, plans, and forms that you can customize with your information – which is private so that nobody
Done
Internet
100%
Start
Removable Disk (E:)
Inbox - Microsoft Outlook
Personal coach training...
MumMoodBooster - ...
Microsoft Office Pictur...
4:40

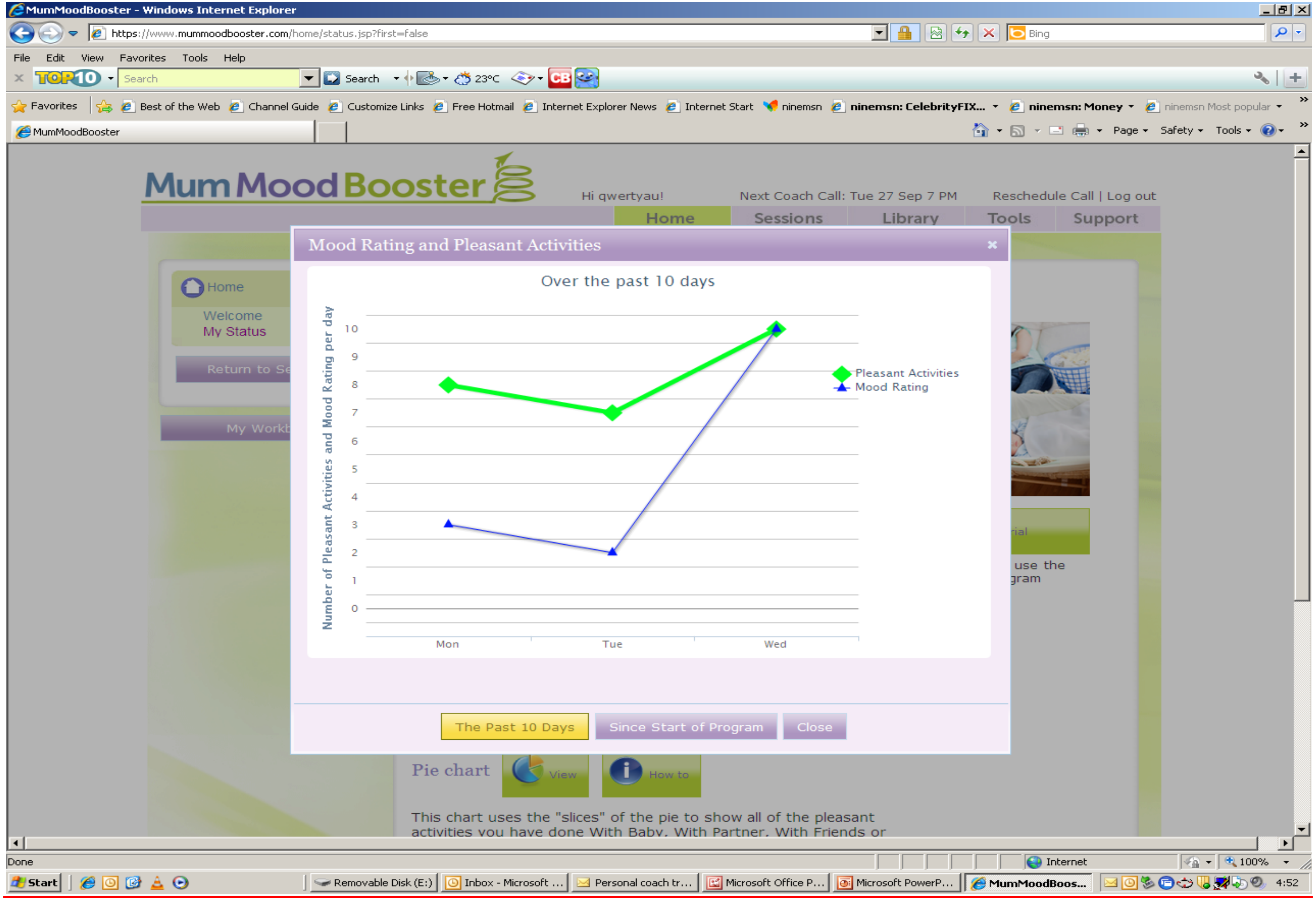

MumMoodBooster - Windows Internet Explorer
https://www.mummoodbooster.com/home/status.jsp?first=false
File Edit View Favorites Tools Help
Mum Mood Booster
Hi qwertyau!
Next Coach Call: Tue 27 Sep 7 PM
Reschedule Call | Log out
Home Sessions Library Tools Support
Home
Welcome
My Status
Mood Rating and Pleasant Activities
Over the past 10 days
Number of Pleasant Activities and Mood Rating per day
Pleasant Activities
Mood Rating
Mon
Tue
Wed
The Past 10 Days
Since Start of Program
Close
Pie chart
View
How to
This chart uses the "slices" of the pie to show all of the pleasant
activities you have done With Baby, With Partner, With Friends or
Done
Internet
100%

My Workbook

Now type your pleasant activities in the list using the four major headings. You can also click the **List>** button to the right side of each blank line to see ideas that can help get you started.

You may find some headings easier than others. This is normal but we encourage you to choose from all headings if you possibly can.

More Fun Activities

Tutorial

Choosing pleasant activities

My Pleasant Activities List

Print ?

With Baby

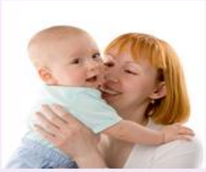

1 Play with toy — List >
2 Stroll with baby — List >
3 Take Baby and Me class — List >
4 Sing to baby — List >
5 Tickle baby — List >

By Myself

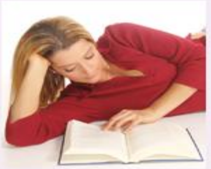

1 Take a walk outside — List >
2 Do yoga — List >
3 Relax — List >
4 Read — List >
5 Go out for coffee — List >

With Friends / Family

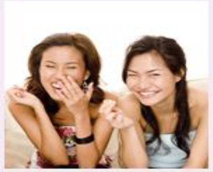

1 Out for dinner/drinks/desser — List >
2 Use Internet — List >
3 Out to movie/play/concert — List >
4 Get a manicure/pedicure — List >
5 Have a picnic — List >

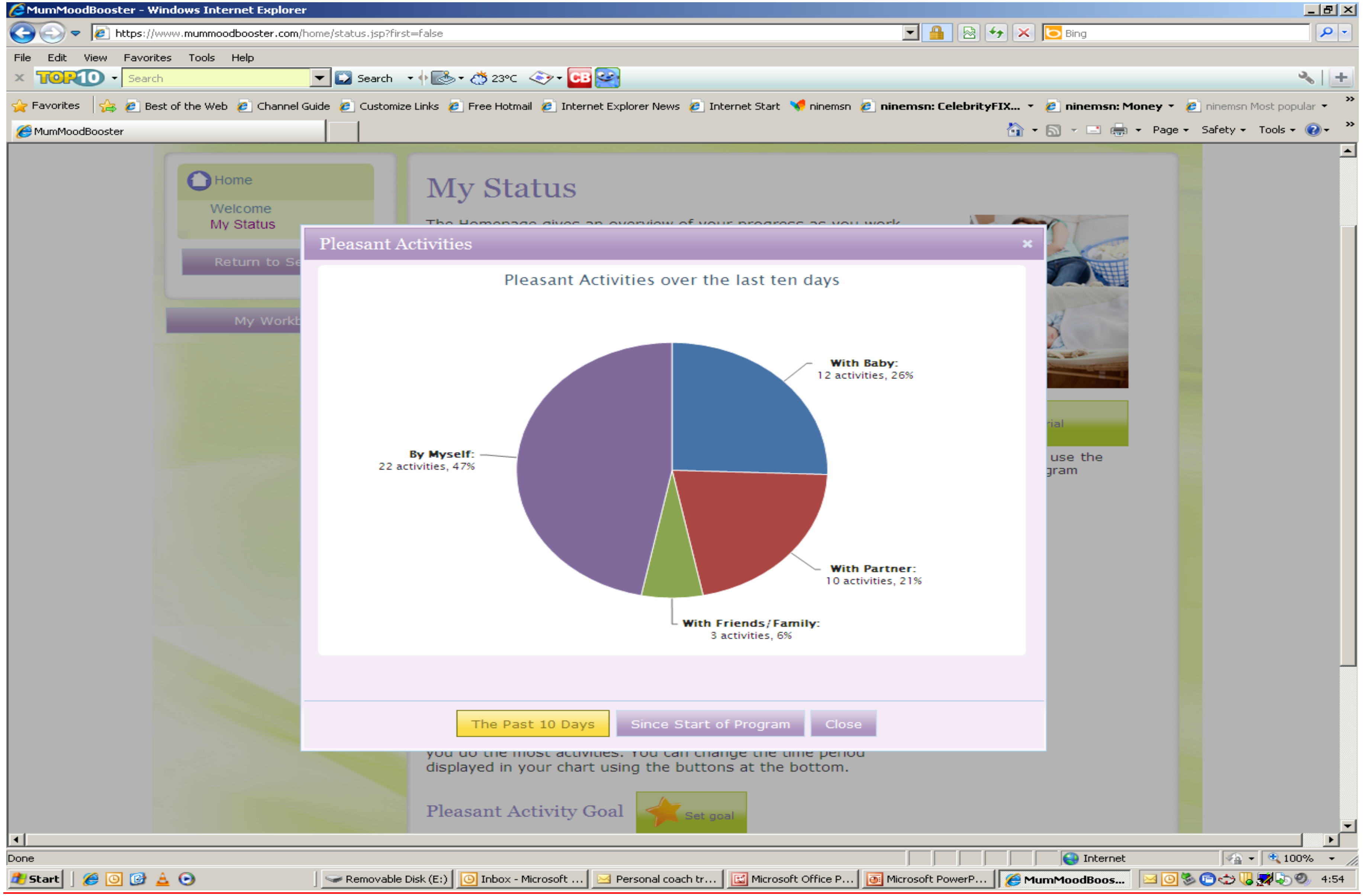

Pleasant Activities
Pleasant Activities over the last ten days
With Baby: 12 activities, 26%
By Myself: 22 activities, 47%
With Partner: 10 activities, 21%
With Friends/Family: 3 activities, 6%
The Past 10 Days
Since Start of Program
Close
My Status
Pleasant Activity Goal
Set goal

MumMoodBooster - Windows Internet Explorer

https://www.mummoodbooster.com/library/getting_support_f.jsp

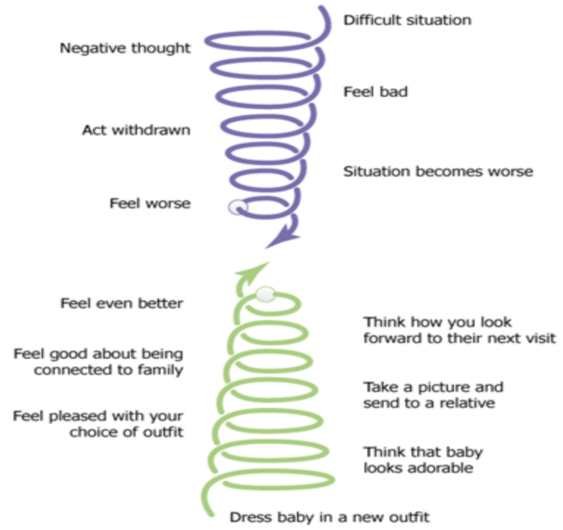

Downward Mood Spiral

A downward spiral is when we do things that make us feel worse and worse. You learned how to catch yourself before you react automatically and end up falling into a downward mood spiral. The goal is to interrupt this pattern so you can choose how you want to react.

Upward Mood Spiral

Focusing more on positive thoughts and doing pleasant activities can help you stop a downward mood spiral and actually improve your chances to have an upward mood spiral. This can lead you to feel better about yourself, your baby, your partner, and the things around you.

- ⊞ My Downward Mood Spiral
- ⊞ Managing My Stress & Anxiety
- ⊞ Increasing My Pleasant Activities
- ⊞ Managing My Negative Thoughts
- ⊞ Increasing My Positive Thoughts
- ⊞ Strategies for Planning Ahead
- ⊞ Personal Progress

Print Close

NEXT
